# Meetings of Societies

**Published:** 1950-07

**Authors:** 


### Meetings of Societies

### BRISTOL MEDICO-CHIRUGICAL SOCIETY

THE Fourth Meeting of the session was held in the University on Wednesday, January 11th, 1950: the President, Dr. H. J. Orr-Ewing, in the Chair. Dr. R. C. Browne, Nuffield Professor of Industrial Health in the University of Durham, gave an address on "**The Work of a Department of Industrial Health**", (p. 67).

The Fifth Meeting was held on February 8th. Lord Moran gave an address on "**The National Service Army**", devoted largely to the philosophy of courage and of morale.

The Sixth Meeting was held on March 8th. Dr. G. D. Kersley gave an address on "**American Rheumatology and Recent Advances in connection with Cortisone**".

The Seventh Meeting was held on April 12th. Mr. Wilfred Adams described the results of "**Nephrectomy for Tumour**": his paper will appear shortly in the *British Journal of Surgery*. Dr. Curwen then gave an address on "**The Value of Radiotherapy in the Treatment of Metastasis in Bone from Carcinoma of the Breast**", (p. 88).

The last meeting of the session was held on May 10th. Dr. D. H. Davies, Mr. A. Miller and Mr. G. L. Alexander opened a discussion on "**The Surgical Treatment of Hypertension**". We hope to publish their remarks in our next issue. The Editor of the *Journal* asked members to indicate whether they were interested in Reviews published in the *Journal* and wished them to be continued. The question had been raised at the March meeting and the financial aspect briefly discussed in April: during three years the average cost of setting type for reviews exceeded the published price of the books reviewed. Although the subject had appeared on the agenda-paper, only eight readers indicated any interest in "Reviews of Books".

### The Clinical Society of Bath

*October Meeting.* Dr. R. H. G. H. Denham, M.O.H. for Bathavon, introduced a discussion on "**General Practice versus Public Health, a commentary on both**". His principal points were the value of some form of supervised introduction of the newly-qualified practitioner into general practice; the importance of his having a full idea of the functions and help which the M.O.H. could give to him; the need in public health work of not employing the most junior M.O. entirely on the routine examination of school-children and of increased liaison between the M.O.H., G.P. and hospital staffs.

*November Meeting.* Dr. G. Kersley discussed "**Impressions of Rheumatology in America and the advances in Endocrine Therapy,**" (p. 82).

*December Meeting.* Mr. S. Glaser presented a paper on "**The Surgery of Hypertension**". He defined the grades of hypertension and main causal conditions to stress the need for accurate assessment before deciding that sympathectomy might reasonably yield lasting benefit.

*January Meeting.* Dr. R. G. Gordon dealt with the many problems of "**Subacute Rheumatism**", stressing particularly the difficulty in classifying so widely spread a condition and the differential diagnoses. He closed with a very neat résumé of the recent views and literature on the subject as a whole.

*February Meeting.* The following questions were discussed: Is fibrositis a clinical entity? Are chronic inflammatory lesions more common since the advent of penicillin? What is the psychiatric abnormality which causes psychiatrists to be interested in psychiatry? Is agorophobia amenable to treatment? Will the present form of maternity service lead eventually to the abandonment of obstetrics as an integral part of G.P.?

*March Meeting.* Dr. J. Macquistan: " **Experiences and Changes in twenty-five years of Medical Practice** ".

*April Meeting.* The President, Dr. W. Love, introduced as the Guest of the Society Professor Ian Aird, who gave a talk on " **A British Impression of American Medicine and Medical Schools** ".

### Cornwall Clinical Society

On Thursday, May 4th, the retiring President, Mr. M. R. Sheridan, gave an address on " **Historical Aspects of Otorhinolaryngology** ". Mr. G. P. O'Donnell was installed as President for the ensuing session.

### Plymouth Medical Society

This Society, under the presidency of Mr. Edric Wilson, had three exceptionally good lectures during the past season. Mr. V. E. Negus described the protection of the Air Passages and showed his beautiful film of ciliary action of the pharynx of the frog. Professor C. Bruce Perry's lecture on the " **Diagnosis and Treatment of Acute Rheumatism** " was a classic example of what a lecture to a provincial medical society should be. Dr. G. S. Todd, gave us the benefit of his experience with Streptomycin in Pulmonary Tuberculosis.

### South-Western Laryngological Association

A meeting was held at The Royal Cornwall Infirmary, Truro, on Saturday, April 1st, Mr. W. H. Bradbeer, President, in the chair. Twelve cases were shown and provoked a keen discussion: for the most part they illustrated results obtained by operation for various conditions. Mr. G. R. Scarff was elected President for the ensuing session. The next meeting will be on Saturday, October 14th at Exeter.

### South-West Region Radiological Society

On May 13th, 1950, a meeting was held of the British Institute of Radiology and South-West Region Radiological Society at the Taunton & Somerset Hospital; this was the first occasion on which these societies have met in Taunton. Dr. McAlley, D.M.R.E., described radiological work in cottage and country hospitals. A series of interesting cases was shown including " **Gumma of Stomach** " and a series of Pancoast's tumours.

### West Somerset Medical Club

On June 1st, Dr. Orr-Ewing addressed the Club at the Castle Hotel, Taunton, on " **Medicine and the Arabs** ". Several very amusing anecdotes were told and the history of Arab medicine was discussed at great length. The lecture was greatly appreciated by all present.

### Wessex Rahere Club

At the annual dinner of the Club, which is open to all old Bart's men resident in the West Country, it was agreed that an additional dinner be held each year away from the main Bath–Bristol area to enable more distant members to attend. A dinner was therefore arranged at the County Hotel, Taunton, on April 29th, and a very satisfactory attendance secured. The Chairman, Professor Brocklehurst, presided and Mr. J. B. Hume journeyed from Bart's to be present as Guest of Honour. In his reply to the toast of " The Hospital ", he outlined the situation at the Hospital, the gradual return of the main departments from their war dispersal areas, the rebuilding and re-organizing which was taking place, staff changes, and the effect of woman graduates on decennial and clubs such as the Wessex Rahere.

### British Medical Association

#### Bath, Bristol and Somerset Branch

A meeting of the Branch was held at the Chamberlain Hall, Locking Road, Weston-s.-Mare, the President, Dr. Percy Phillips, in the Chair. Professor W. F. Gaisford, F.R.C.P., from the Department of Child Health, University of Manchester, gave an address on **" Medicine and Children".**

Wednesday, June 28th. Clinical meeting at Bristol General Hospital.

#### Bristol Division

*Officers:* Chairman, Dr. G. E. F. Sutton; Chairman elect, Dr. T. Milling; Secretaries, Drs. H. M. Golding, W. H. Hayes; Treasurer, Dr. C. Dix.

#### Newport Division

January 24th, 1950. Dr. Charles Hill on **" Current Affairs ".**

February 9th. Clinical meeting at Royal Gwent Hospital. Kartagener's syndrome and kinked carotid (Dr. Dipple); oesophagectomy (Mr. H. Roberts), cases of Smithwick's operation (Mr. V. Jones) and other cases: a film illustrating Rammstedt's operation for pyloric stenosis by Mr. Rice Edwards.

March 23rd. Mr. Alec Bourne on **" The Endocrines in Gynaecology ".**

April 16th. Annual General Meeting: Dr. Bird was elected President and Drs. Bernard Thomas and Trevor Bryant, Joint Secretaries.

